# Multifunctional extracellular vesicles and edaravone-loaded scaffolds for kidney tissue regeneration by activating GDNF/RET pathway

**DOI:** 10.1186/s40580-024-00450-5

**Published:** 2024-10-26

**Authors:** Seung Yeon Lee, Jeong Min Park, Won-Kyu Rhim, Eun Hye Lee, Sang-Hyuk Lee, Jun Yong Kim, Seung-Gyu Cha, Sun Hong Lee, Boram Kim, Dong-Youn Hwang, Seungsoo Rho, Tae-Keun Ahn, Bum Soo Kim, Dong Keun Han

**Affiliations:** 1 Department of Biomedical Science, CHA University, Seongnam, Gyeonggi-Do 13488 Republic of Korea; 2 Joint Institute for Regenerative Medicine, Kyungpook National University, Jung-Gu, Daegu, 41944 Republic of Korea; 3 Department of Microbiology, School of Medicine, CHA University, Seongnam, Gyeonggi-Do 13496 Republic of Korea; 4 Department of Ophthalmology, CHA Bundang Medical Center, CHA University, 59 Yatap-Ro, Bundang-Gu, Seongnam, Gyeonggi-Do 13496 Republic of Korea; 5 Department of Orthopedic Surgery CHA Bundang Medical Center, CHA University, Seonnam, Gyeonggi-Do 13496 Republic of Korea

**Keywords:** Kidney regeneration, Scaffold, Edaravone, Extracellular vesicle, SDF1α

## Abstract

With the severity of chronic kidney disease worldwide, strategies to recover renal function via tissue regeneration provide alternatives to kidney replacement therapy. To exclude side effects from direct cell transplantation, extracellular vesicles (EVs) are great substitutes representing paracrine cell signaling. To build three-dimensional structures for implantation into the 5/6 nephrectomy model by incorporating bioactive materials, including multifunctional EVs (mEVs), porous PMEZE/mEV scaffolds were developed in combination with edaravone (EDV; E) and mEV based on PMEZ scaffolds with PLGA (P), MH-RA (M), ECM (E), ZnO-ALA (Z). The oxygen free radical scavenger EDV was incorporated to induce tubular regeneration. mEVs were engineered to serve regenerative activities with a combination of two EVs from SDF-1α overexpressed tonsil-derived mesenchymal stem cells (sEVs) and intermediate mesoderm (IM) cells during differentiation into kidney progenitor cells (dEVs). mEVs displayed beneficial effects on regeneration by facilitating migration and inducing differentiation of surrounding stem cells, and EDV improved kidney function by regulating the GDNF/RET pathway and their downstream genes. The promotion of MSC recruitment was confirmed with sEV particles number dependently, and the regulation of the GDNF/RET pathway by the effect of EDV and its enhanced effect by mEVs were elucidated using in vitro analysis. The regeneration of tubules was additionally demonstrated through the increased expression of aquaporin-1 (AQP-1) and cadherin-16 (CDH16) for proximal tubules, and calbindin and PAX2 for distal tubules in the renal defect model. With these, structural regeneration and functional recovery were achieved with kidney regeneration in the 5/6 nephrectomy mice model.

**Graphical abstract:**

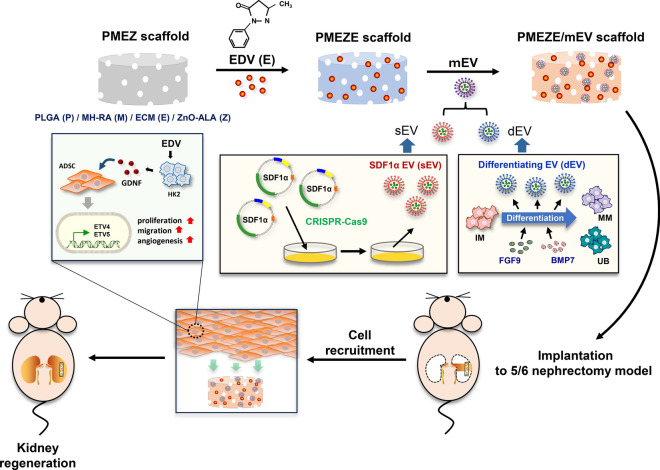

**Supplementary Information:**

The online version contains supplementary material available at 10.1186/s40580-024-00450-5.

## Introduction

Chronic kidney disease (CKD) is a complex disease that occurs due to functional damage to the kidneys and is accompanied by complications such as increased cardiovascular disease, anemia, altered mineral metabolism, and metabolic acidosis [1–3]. In general, CKD is caused by genetic, metabolic, immunological, and vascular factors, of which metabolic and vascular factors are most common [4]. CKD progression is characterized by a progressive accumulation of kidney fibrosis caused by excessive extracellular matrix (ECM) deposition and myofibroblast accumulation. Concurrently, glomerulosclerosis, tubular atrophy, and irreversible damage to parenchymal cells lead to a progressive deterioration of kidney function [5, 6]. Although dialysis helps patients with end-stage renal disease (ESRD) survive, kidney transplantation is the only method for complete treatment. However, kidney transplantation requires continuous immunosuppressant medication. Despite this, ~ 40% of patients are expected to lose their transplant function or die within 10 years, posing difficulties for disease treatment [7]. As an alternative to incomplete therapeutic approaches, there has been a focus on tissue engineering and regenerative medicine strategies to reconstruct kidney structures and restore kidney function.

Edaravone (EDV) is a free radical scavenger used to treat ischemic stroke and is a Food and Drug Administration (FDA)-approved drug for amyotrophic lateral sclerosis (ALS) [8, 9]. The drug is a low-molecular-weight antioxidant that targets peroxyl radicals among various reactive oxygen species [10, 11]. Although the ambivalent nature of EDV has been reported in acute kidney injury (AKI) patients with ischemic stroke [12], many studies have supported the protective effects of EDV against many animal models with kidney injury [13], including renal ischemia–reperfusion injury [14], puromycin nephrosis [15], and cisplatin-induced kidney injury [16]. In detail, EDV has a protective effect via glial cell line-derived neurotrophic factor (GDNF)/RET signaling, an activated neurotrophic factor signaling pathway [17]. GDNF, which belongs to the transforming growth factor-β (TGF-β) superfamily, induces the normal growth of the ureteric bud (UB) during kidney development [18]. Li et al. demonstrated GDNF-promoted adipose-derived stem cells (ADSCs) homing to damaged kidneys, enhancing angiogenesis through PI3K/AKT/eNOS pathway [19]. Although several studies have reported an association between EDV and GDNF/RET signaling, the mechanisms during kidney regeneration are unclear.

Among the CXC chemokine subfamily members, stromal cell-derived factor-1 (SDF-1; CXCL12) is the most potent chemoattractive signal and has been identified as a stem cell homing factor [20, 21]. There are six different splicing variants (α, β, γ, δ, ε, and ψ), of which SDF-1α is expressed broadly in several tissues and mediates multiple functions, such as cell adhesion, chemotaxis, and survival [22]. Recent studies have demonstrated that SDF-1 is a key chemokine and homing factor for stem cells, promoting their migration to damaged sites [23, 24]. Togel et al. showed mobilization of renal SDF-1 signals and homing of C-X-C chemokine receptor 4 (CXCR4)-positive cells to the kidney after ischemic injury. They asserted the important role of SDF-1 as a significant signal for kidney recovery [25]. Clustered regularly interspaced short palindromic repeats (CRISPR)/CRISPR-associated protein 9 (CRISPR/Cas9) is an effective gene-editing technology for engineering cells to express large amounts of protein of interest in a microbial adaptive immune system that cleaves foreign genetic material using RNA-guided nucleases [26]. Using the SDF-1-overexpressed plasmid vector already constructed via CRISPR/Cas9-mediated knock-in gene editing [27], tonsil-derived mesenchymal stem cells (ToMSCs) were engineered with the SDF-1α gene using the CRISPR-Cas9 system to isolate extracellular vesicles (EVs) for migration of surrounding stem cells to injured tissues.

Stem cell-derived EVs have great potential in kidney regeneration due to their inhibitory activities for damage progression and fibrosis of injured kidney tissues [28, 29]. EVs are naturally occurring 40–150 nm vesicles that communicate between cells by encasing proteins, lipids, metabolites, and genetic information in lipid bilayers [30–33]. Soluble cytokine and inflammatory mediator signaling pathways are key participants in kidney disease pathogenesis. EVs may play critical roles in repairing and regenerating kidney tissue damages by mediating interneuron communication [34, 35]. Nevertheless, EVs alone are insufficient for tissue regeneration, especially under challenging conditions due to their limited inductive capacity [36]. Engineered EVs have garnered a lot of interest in overcoming insufficient functionalities of native EVs and have demonstrated remarkable promises as a therapeutic platform in tissue regenerative medicine [37, 38]. A previous study developed biodegradable scaffolds loaded with various engineered EVs to achieve composite tissue regeneration effects for CKD treatment [39, 40]. Interestingly, the integration of engineered EVs with drugs showed synergistic effects on functional kidney tissue regeneration and restoration by enhancing cell proliferation, promoting angiogenesis, and alleviating fibrosis and inflammatory responses. However, improved strategies are still needed due to the lack of approaches to fully utilize surrounding cells and biofunctionalized EVs for more practical kidney regeneration.

**Fig. 1:**
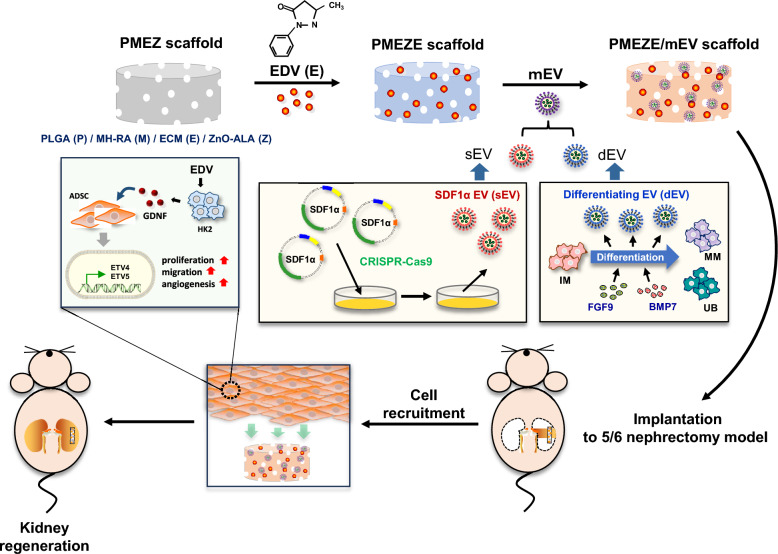
Schematic illustration of the fabrication of functionalized scaffolds for kidney tissue regeneration. Multiple functional components, such as MH-RA (M), ECM (E), ZnO-ALA (Z), EDV (E), and engineered EVs (mEVs = sEVs + dEVs), were combined to generate a functionalized PMEZE/mEV scaffold and its topical application to the 5/6 nephrectomy mice model leading to improved kidney regeneration

In this study, multifunctional scaffolds were developed by mixing EDV with engineered multifunctional EVs (mEVs) containing SDF-1α-overexpressed ToMSCs (sEVs) and kidney differentiating EVs (dEVs) isolated during intermediate mesoderm (IM) differentiation into kidney progenitor cells, metanephric mesenchyme, and UB for kidney regeneration (Fig. 1). It was hypothesized that SDF-1α could recruit surrounding stem cells to injured tissue area and that EDV and differentiation-related factors could enhance the regenerative capacity of these stem cells via the GDNF/RET signaling pathway in damaged kidney tissue. It was envisioned that a multifunctional scaffold construct would provide great opportunities for functional kidney restoration by promoting kidney tissue regeneration.

## Materials and methods

### Materials

Poly(D,L-lactic-co-glycolic acid) (PLGA; LA/GA = 50:50, MW 40,000) was obtained from Evonik Ind. (Essen, Germany). Magnesium hydroxide [Mg(OH)_2_; MH-RA], zinc oxide (ZnO), retinoic acid (RA), heparin sodium salt, and EDV were purchased from Sigma-Aldrich (St. Louis, MO, USA). Ricinoleic acid and DL-α-lipoic acid (ALA) were purchased from TCI Product (Tokyo, Japan). Anti-CD63, TSG101, and Apo-A1 primary antibodies were purchased from Santa Cruz Biotechnology (Santa Cruz, CA, USA). Anti-rabbit IgG and anti-mouse IgG secondary antibodies were purchased from Cell Signaling Technology (Danvers, MA, USA). Enzyme-linked immunosorbent assay (ELISA) kits were purchased from R&D Systems (Minneapolis, MN, USA). Recombinant human fibroblast growth factor 9 (FGF9) and bone morphogenetic protein 7 (BMP7) were purchased from Peprotech (Rocky Hill, NJ, USA).

### Cell culture

sEVs were kindly provided by Dr. Dong-Youn Hwang [27] and were grown in CellCor™ CD MSC medium (Xcell Therapeutics, Seoul, Korea) supplemented with 1% antibiotic–antimycotic solution (A/A; Gibco, Grand Island, NY, USA) to obtain sEVs. Human pluripotent stem cell-derived IM was obtained from Dr. Dong Ryul Lee, and their differentiation conditions were described in previous studies [40]. Briefly, FGF9 (200 ng/mL), BMP7 (50 ng/mL), RA (30 ng/mL), and heparin (1 μg/mL) were added to the medium and replaced every 2 days for 12 days to induce differentiation IM into kidney progenitor cells. Human renal proximal tubular epithelial cell (HK2) was obtained from the Korean Cell Line Bank (Seoul, Korea) and cultured in RPMI 1640 medium (Gibco) supplemented with 10% fetal bovine serum (FBS; Hyclone Laboratories, Logan, UT, USA) and 1% A/A. Human umbilical vein endothelial cells (HUVECs) were obtained from Lonza (Basel, Switzerland) and cultured with EGM-2 endothelial cell growth medium-2 Bullet Kit (Lonza). ADSCs were obtained from Lonza and cultured in Dulbecco’s modified Eagle’s medium with low glucose (Cytiva, Seoul, Korea) supplemented with 10% FBS and 1% A/A. All cell types were cultured at 37 °C in a humidified atmosphere containing 5% CO_2_.

### Isolation and characterization of EVs

EV isolation and characterization were performed as optimized previously [40]. Two EVs, sEVs and dEVs, were isolated from SDF-1α-overexpressing ToMSCs and kidney differentiating IM cells, respectively, using a tangential flow filtration system (KR2i TFF; Repligen, Waltham, MA, USA). To prepare conditioned media (CM) for EV isolation, cell culture supernatants were collected every 2 days for 12 days. The supernatants were centrifuged at 1300 rpm (3 min at room temperature) and filtered using a 0.22 μm vacuum filter/storage bottle system to remove larger particles than EVs. EVs were isolated using the TFF system with a 500 kDa molecular weight cutoff filter. The collected EVs were resuspended in phosphate-buffered saline (PBS) solution and stored at − 80 °C. EV sizes and concentrations of EVs were measured by nanoparticle tracking analysis (NTA) using MONO ZetaView^®^ with a 488 nm scatter mode (PMX-120, Particle Metrix, Meerbusch, Germany), and morphology was observed by transmission electron microscopy (TEM; H-7600, 80 kV; Hitachi, Tokyo, Japan) after negative staining procedures.

### Western blot analysis

Cells were resuspended in radioimmunoprecipitation assay (RIPA) buffer with a protease inhibitor. An equal amount of EVs (1 × 10^9^ particles) was separated using 12% sodium dodecyl sulfate–polyacrylamide gel electrophoresis and transferred onto nitrocellulose membranes in transfer buffer. The blots were blocked for 1 h at room temperature with 5% skim milk and incubated overnight at 4 °C with TSG101, CD63, and Apo-A1 antibodies at 1:200 dilution. After washing with Tris-buffered saline (TBS) solution containing 0.05% Tween 20, the blots were incubated for 1 h at room temperature with anti-rabbit IgG or anti-mouse IgG horseradish peroxidase-linked secondary antibodies (1:2000). Protein bands were exposed via enhanced chemiluminescence solution (ECL; GE Healthcare, Milwaukee, WI, USA) and visualized using ChemiDoc™ XRS + with ImageLab (Bio-Rad, Hercules, CA, USA).

### ELISA

To evaluate SDF-1α levels in EVs, a commercially available Quantikine™ ELISA kit (R&D Systems) was used. All experimental steps were performed according to the manufacturer’s instructions. Absorbance was determined at a wavelength of 450 nm using a microplate reader (Molecular Devices, Silicon Valley, CA, USA).

### Fabrication and characterization of scaffolds

The ice particle leaching technique was utilized to prepare multifunctional polymer scaffolds, including poly(lactic-co-glycolic acid) (PLGA; P), magnesium hydroxide (MH-RA; M), extracellular matrix (human adipose tissue-derived extracellular matrix, ECM; E, Hans Biomed Inc.), DL-α-lipoic acid-conjugated zinc oxide (ZnO-ALA; Z), and edaravone (EDV; E). Briefly, the PLGA (0.25 g), MH-RA (15 wt%), ECM (20 wt%), ZnO-ALA (10 wt%), and EDV (300 μM) were dissolved and mixed in dichloromethane (1.92 mL). The optimal EDV concentration was investigated by comparing the ADSC survival rate according to the EDV concentration (Fig. S1). After the freeze-drying process to eliminate volatile organic components, 1 × 10^9^ particles of sEVs and 5 × 10^8^ particles of dEVs were loaded onto hydrated scaffolds with a stabilizing process for 24 h. To observe the distribution of mEVs (sEVs + dEVs) in the scaffolds, DiO (Thermo Fisher, Waltham, MA, USA)-labeled mEVs were loaded into composite scaffolds and observed with a confocal laser microscope (LSM880, Carl Zeiss, Jena, Germany). The release profile of DiO-labeled EVs was quantified by a microplate reader at a wavelength of 483 nm. The morphology of the scaffolds was observed using field emission-scanning electron microscopy (FE-SEM; Sigma, Carl Zeiss, Jena, Germany) with an operating voltage of 5 kV. All scaffolds were coated with gold for 30 s using a sputtering apparatus before imaging. Thermogravimetric analysis (TGA; PerkinElmer, Waltham, MA, USA) was performed to measure the mass of inorganic components in the scaffolds, employing a heating rate of 10 °C/min from 30 to 800 °C. The chemical bonding of the surface was determined by attenuated total reflectance-Fourier transform infrared (ATR-FTIR; Sigma, Carl Zeiss, Oberkochen, Germany) from 650 to 4000 cm^−1^ and scan speed with 0.2 cm/s. The mechanical strengths of scaffolds were evaluated using a universal testing machine (UTM; Instron, Norwood, MA, USA). The compressive modulus was calculated from the stress–strain curve by the average slope of 5 to 10% strain. The pH changes were measured by a pH meter (Mettler Toledo, Columbus, OH, USA) in 1 mL PBS solution at 37 °C for 28 days. The amount of released EDV was quantified using high-performance liquid chromatography (HPLC; Vanquish VC-P20-A; Thermo Fisher Scientific, Marietta, OH, USA) with an Agilent Eclipse column (5 μm, 4.6 × 150 mm C18) at 40 °C. The mobile phase was composed of water and acetonitrile (50:50, v/v) and flowed at a flow rate of 1 mL/min. The profile was observed at a wavelength of 243 nm. To evaluate the degradation rate of scaffolds, each scaffold was individually immersed in 1 mL PBS solution and incubated at a 37 °C shaker with 150 rpm/min. At every defined time interval, the percentage of mass loss was calculated after the samples were lyophilized.

### Cell viability assay

Cell viability of HK2 or ADSCs was obtained using the Cell Counting Kit-8 (CCK-8; Dojindo, Japan). A working solution with 10% CCK-8 reagent was added to each well and incubated at 37 °C for 2 h. Absorbance was measured at 450 nm using a microplate reader.

### Migration assay in the Transwell system

For migration assay, 5 × 10^8^ cells were transferred from the cell culture plate into the upper chamber of an 8 μm pore size membrane in a Transwell cell culture system (SPL Life Sciences, Gyeonggi-do, Korea). Media containing scaffolds were added to the lower chamber. After 24 h incubation, cells remaining in the upper membrane were removed with cotton applicators. Cells that migrated through the membrane were stained with methanol and 1% crystal violet and monitored using an optical microscope (CKX53; Olympus, Tokyo, Japan).

### Wound healing assay

HK2 cells were initially seeded on the 6-well plate at a density of 1.5 × 10^5^ cells/well. After cells adhered to the well for 16 h, a straight scratch was made on the cell monolayer using a sterile 1 mL pipette tip. The scratched areas were immediately and gently rinsed twice with PBS solution (Welgene, Gyeongsan-si, Korea). Cells were incubated at 37 °C for 16 h with PMEZ, PMEZE, and PMEZE/mEV scaffolds, respectively. After incubation, wound healing images were observed using an optical microscope. The healing of the wound area was quantified using ImageJ (National Institutes of Health, Bethesda, MD, USA).

### Tube formation assay

The 24-well plate was coated with 300 μL Matrigel basement membrane matrix (Corning, Steuben Country, NY, USA) per well and incubated at 37 °C, 5% CO_2_ to form a gel for 1 h. HUVECs were seeded onto Matrigel-coated wells at the density of 1.2 × 10^5^ cells/well and incubated at 37 °C for 16 h with PMEZ, PMEZE, and PMEZE/mEV scaffolds. Cells were stained with 4 μM calcein AM (Thermo Fisher, Waltham, MA, USA), and the number of branch points and tube length was measured using the angiogenesis plug-in of ImageJ.

### Real-time quantitative polymerase chain reaction (RT-qPCR) of in vitro samples

For RT-qPCR assays, total cellular RNA was extracted from cells using the AccuPrep® Universal RNA Extraction Kit (Bioneer, Daejeon, Korea) according to the manufacturer’s instructions. Reverse transcription was performed using PrimeScript™ RT reagent kit (Takara, Shiga, Japan). For RT-qPCR, primer sequences were designed as described previously [41]. RT-qPCR was performed with SYBR Green PCR reagents (Applied Biosystems, Foster City, CA, USA) using QuantStudio 3 (Applied Biosystems). For data quantification, the 2^−ΔΔCt^ method was applied with 18S rRNA as a reference. Each reaction was performed in three independent experiments.

### Designs for in vivo experiments

All protocols for in vivo experiments were approved by the Institutional Animal Ethics Committee of Yeungnam University College of Medicine (YUMC-AEC2023-028). Six-week-old female ICR mice were purchased from Jung Ang Lab Animal, Inc. (Seoul, Korea) and randomly divided. To establish the 5/6 nephrectomy mouse model, both kidneys were dissected 2 to 3 mm bipolarly each (apical and inferior poles). Two and 8 weeks after scaffold implantation in the right kidney, mice were sacrificed and kidney tissues were retrieved for subsequent analysis. Animals were anesthetized with 3 to 4% isoflurane for induction and 1 to 3% for maintenance.

### RT-qPCR of in vivo samples

The Maxwell^®^ RSC simply RNA cell kit was used to isolate RNA from kidney tissues by operating the Maxwell™ 16 instrument (Promega, Madison, WI, USA). Isolated RNA (1 μg) was used to synthesize cDNA with the GoScript™ Reverse Transcription Mix (Promega) according to the product protocols. RT-qPCR was performed in the StepOnePlus™ Real-Time PCR System (Applied Biosystems) using LUNA NEB SYBR Green Master Mix (NEB, MA, USA). The primer sequences were designed as described previously report [40].

### Histological and functional analyses

All collected kidney tissues were fixed in 10% formalin and embedded in paraffin. Tissue sections were cut into 4 μm thicknesses and applied on coated slide glasses. Hematoxylin and eosin (H&E), periodic acid-Schiff (PAS), and Masson’s trichrome (MT) staining were used for general histology, glomerulosclerosis, and fibrosis, respectively. Slides were examined under light microscopy. For immunohistochemistry, slides were deparaffinized and hydrated by xylene and ethanol. Citrated buffer solution was used for antigen retrieval before blocking with 5% bovine serum albumin solution. The primary antibodies (1:100) were applied for 18 h at 4 °C. Fluorescein isothiocyanate-conjugated secondary antibody was applied for 2 h at room temperature. The slides were mounted with the 4′,6-diamidino-2-phenylindole (DAPI) staining medium (Vector Laboratories, Burlingame, CA, USA) and examined under fluorescence microscopy.

### Statistical analysis

Data were analyzed using GraphPad Prism version 9 (GraphPad Software, San Diego, CA, USA). Results are expressed as the mean ± standard deviation (SD). Unpaired *t*-tests or one-way analysis of variance (ANOVA), followed by Tukey’s multiple comparison post-test, were used to analyze group differences. For all experiments, statistical significance was defined as *p < 0.05, **p < 0.01, ***p < 0.001, and ****p < 0.0001 for three biological replicates.

## Results and discussion

### Generation of sEVs

**Fig. 2:**
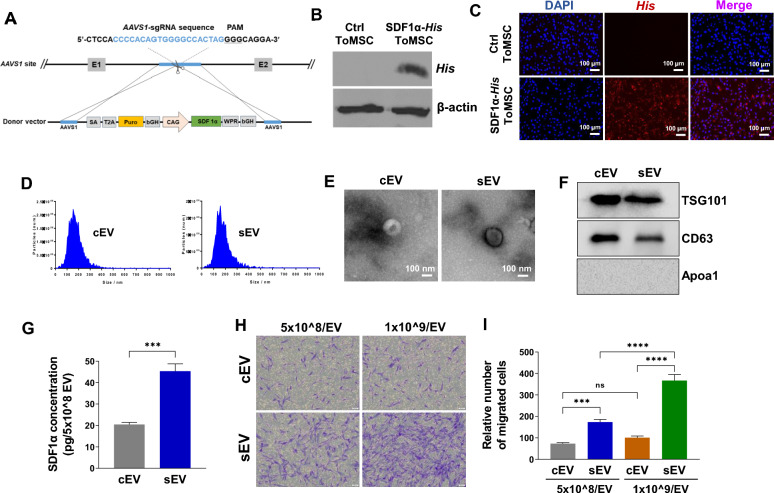
Characterization of sEVs. **A** CRISPR/Cas9-mediated knock-in of SDF-1α into a safe-harbor site (AAVS1) on the ToMSC chromosome. **B** Evaluation of SDF-1α expression by Western blotting and **C** immunochemistry. Scale bars = 100 μm. **D** NTA to compare the size distribution of cEVs and sEVs. **E** TEM analysis to observe the structure and size of purified cEVs and sEVs. Scale bars = 100 nm. **F** Western blot analysis for TSG101, CD63, and Apoa1 protein expression in cEVs and sEVs. (G) ELISA to confirm SDF-1α overexpression in sEVs. **H** Migration ability of sEV-treated ADSCs determined using the Transwell assay. Scale bars = 200 μm. **I** Quantitative analysis of the cell migration. ***p < 0.001; ****p < 0.0001; ns, no significant difference

Chemokine receptors play a crucial role in the migration of injected cells to the site of injury; therefore, manipulating these receptors can decrease the unwanted migration of transplanted cells and facilitate their targeted migration to the intended site [42]. Among them, SDF-1 and its main receptor CXCR4 play an important role in stem cell motility and development [43]. In a previous study, the CRISPR/Cas9 system was utilized to engineer cells for inducing stem cell recruitment by overexpressing SDF-1α under the CAG promoter [27]. The CRISPR/Cas9 delivery system is suitable for engineering EVs due to several advantages over viruses and other vectors, such as protection, safety, capacity, penetration capability, targeting capability, and modification potential [44]. Gong et al. demonstrated that SDF-1 overexpression in MSC-derived EVs inhibits autophagy of ischemic myocardial cells and promotes the microvascular production of endothelial cells. However, the role of SDF-1 overexpression in ToMSC-derived EVs in regulating kidney regeneration in vivo has not been explored [45]. To manipulate EVs with SDF-1α overexpression, the CRISPR/Cas9 system was used to insert the SDF-1α gene into ToMSCs by integrating into the safe harbor site, the adeno-associated virus integration site 1 (AAVS1) locus, of the ToMSC chromosome (Fig. 2A). ToMSCs that were genetically engineered by SDF-1α overexpression were verified by detecting SDF-1α-His tag via Western blot and immunofluorescence analyses (Fig. 2B and C). SDF-1α-His-overexpressed ToMSCs displayed a higher amount of His protein expression than the negligible amount in control ToMSCs. Control EVs (cEVs) and SDF-1α EVs (sEVs) were serially isolated from control ToMSCs and sEVs, respectively. Both EVs showed similar particle mean size and size distribution in NTA analysis (Fig. 2D), and there were no clear difference as typical cup-shaped and spherical morphologies in TEM images (Fig. 2E). Moreover, the concentrations of isolated EVs were similar at 10^8^ particles/mL. Western blotting showed that both EVs showed the expression of TSG101 and CD63, generally recognized as EV-positive markers (Fig. 2F), whereas the expression of the representative negative marker, Apoa1, was not detected. To confirm SDF-1α overexpression in sEVs, the amount of SDF-1α protein was quantified by ELISA, and the SDF-1α concentration was higher in sEVs (45.3 pg/5 × 10^8^ EVs) compared to cEVs (20.5 pg/5 × 10^8^ EVs; Fig. 2G). ADSCs exhibit several attractive characteristics compared to other MSCs, including abundant numbers, accessibility in cell culture, steady function, and less immunological rejection [46, 47]. Migration and tropism to the injured tissue of ADSCs were demonstrated by tracking bioluminescence-labeled ADSCs in the cisplatin-induced AKI model [48]. ADSC migration in Transwell containing two different EVs was demonstrated to verify the MSC migration ability of SDF-1α in EVs. A Transwell migration assay showed that ADSC migration significantly increased in wells containing sEVs compared to wells containing cEVs and gradually enhanced as the sEV concentration increased (Fig. 2H and I). These results indicated that SDF-1α in EVs also facilitated ADSC migration at in vitro conditions.

### Characterization of PMEZE/mEV scaffolds

**Fig. 3:**
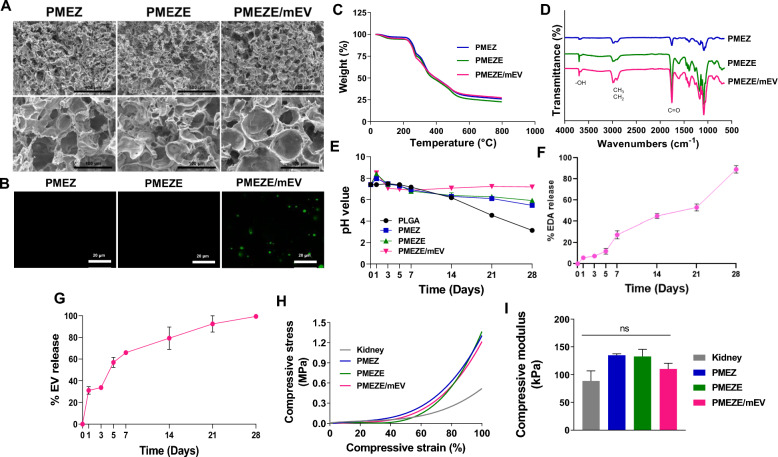
Characterization of functionalized porous scaffolds. **A** SEM images of PMEZ, PMEZE, and PMEZE/mEV scaffolds. Scale bars = 100 μm. **B** Representative confocal images showing DiO-labeled mEV distribution in PMEZ, PMEZE, and PMEZE/mEV scaffolds. Scale bars = 20 μm. **C** TGA thermograms and **D** ATR-FTIR spectra of PMEZ, PMEZE, and PMEZE/mEV scaffolds. **E** Changes in the pH value during in vitro scaffold degradation. Release profile of **F** EDV and **G** mEV during 28 days. **H** Compressive stress–strain curve and **I** compressive modulus at 5 to 10% stress–strain curve. ns: no significant difference

Tissue engineering has remarkable potential in developing novel functional materials for kidney regeneration [49]. Although various polymer-based synthetic scaffolds exist, PLGA has been consistently used in previous studies due to its excellent biocompatibility and biodegradability [39, 40, 50]. In this study, ice particle leaching was employed to fabricate multifunctional PMEZE scaffolds comprising PLGA (P), MH-RA (M), ECM (E), ZnO-ALA (Z), and EDV (E). mEVs, which are combinations of sEVs and dEVs with two distinct functions, were serially seeded into hydrated scaffolds at the density of 1 × 10^9^ EVs/scaffold to manufacture PMEZE/mEV scaffolds. With the help of mEVs, PMEZE/mEV scaffolds were expected to facilitate kidney tissue regeneration by attracting surrounding MSCs and inducing their differentiation into kidney progenitor cells. The uniform and interconnected porous structures with a pore size of 70 to 150 μm were monitored using SEM (Fig. 3A). The highly porous structure facilitates kidney regeneration via cell migration and diffusion of bioactive components between scaffolds and peripheral tissues. The successful introduction of mEV into PMEZE scaffolds was demonstrated by monitoring the distribution of DiO-labeled mEV using confocal microscopy. Well-dispersed fluorescent signals proved mEV incorporation onto the PMEZE scaffold surface (Fig. 3B). TGA was tested to compare the mass ratios of various components in the scaffolds. Although it began to decompose at a lower temperature compared to pure PLGA scaffold [50], there was no significant difference due to the addition of relatively low concentrations of EDV and mEVs compared to PMEZ scaffolds (Fig. 3C). However, EDV addition was demonstrated with ATR-FTIR to further distinguish the chemical composition of different scaffolds. The spectra of multifunctional scaffolds were almost like that of PMEZ, but PMEZE and PMEZE/mEV spectra contained higher intensity of the C = O stretching vibration ~ 1800 cm^−1^ due to C = O stretching in EDV [51] (Fig. 3D). The O–H stretching at 3700 cm^−1^ and C-H stretching at 2927, 2865, and 2844 cm^−1^ were displayed for all scaffolds as proof of the ATR-FTIR spectrum for MH-RA and ZnO-ALA, respectively [52, 53]. The scaffold degradation rate has an undeniable effect on the final fate of the scaffold in vivo and the recovery of injured tissue [54, 55]. Polymer-based scaffolds, especially PLGA, have the problem that the surrounding pH is lowered by acidic byproducts, causing inflammation in peripheral tissues. By introducing MH-RA, which has proven a neutralizing effect in a previous study, the PMEZE/mEV scaffold, which showed a relatively fast degradation time compared to the bare PLGA scaffold, also exhibited a pH neutralizing effect (Fig. 3E; Fig. S2) [50]. Sustained-release substances in the long term are one of the reasons for induced tissue regeneration by implanting a porous scaffold and incorporating bioactive materials. To predict the possibility of long-term tissue regeneration by continuous release of bioactive materials, time-dependent releases of EDV and mEVs were evaluated under physiological conditions (37 °C and pH 7.4). The cumulative EDV release from PMEZE scaffolds reached ~ 53% at 21 days and ~ 89% at 28 days (Fig. 3F). The cumulative release of EVs from the scaffolds showed a sustained-release profile with an initial burst of ~ 31.1% at day 1, 33.7% at day 3, 44.0% at day 5, 51.2% at day 7, 79.3% at day 14, 92.4% at day 21, and 99.4% at day 28 (Fig. 3G). Because mEVs were introduced to the scaffold surface through a simple loading method without using any other chemical bonds, an initial burst release of mEVs occurred, but mEVs were continuously released for 28 days from the scaffolds. The compressive stress of the scaffold with different components was analyzed by a UTM (Fig. 3H). The compressive modulus values were obtained from slope calculations between 5 and 10% of the strain–stress curve. The compressive modulus of PMEZ, PMEZE, and PMEZE/mEV scaffolds was 134.9 ± 3.71, 132.8 ± 10.44, and 110.2 ± 8.35 kPa, respectively. EDV addition did not induce significant changes in compressive modulus, and the PMEZE/mEV value slightly decreased as hydration occurred while introducing mEVs, resembling the actual mechanical strength of the kidney (Fig. 3I). These results indicated that the PMEZE/mEV scaffold is an ideal construct for kidney regeneration.

### In vitro regeneration ability of EDV and mEVs

**Fig. 4:**
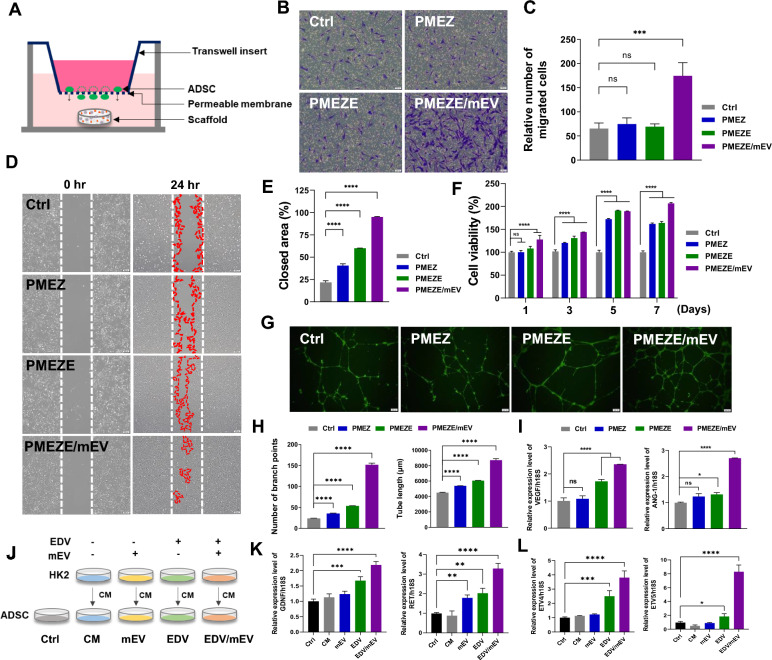
Effect of EDV and mEVs on wound repair and angiogenesis in vitro. **A** Schematic illustration of the ADSC migration assay using a Transwell system. **B** Microscopy images and **C** quantitative analysis of crystal violet-stained ADSCs that migrated from the top to bottom chamber in response to sEV. Scale bars = 200 μm. **D** Representative images and **E** quantitative analysis of wound healing assay. Scale bars = 200 μm. **F** Cell viability of PMEZ, PMEZE, and PMEZE/mEV scaffolds using the CCK-8 assay. **G** Representative images and **H** quantitative analysis of tube formation assay characterizing the number of branch points and tubule length. Scale bars = 200 μm. **I** Gene expression onto scaffolds related to angiogenesis, VEGF and ANG-1, in ADSCs. **J** Schematic illustration for CM collection of HK2 cells and its treatment to ADSCs. **K** GDNF and RET gene expression and their downstream genes, such as ETV4 and ETV5, in ADSCs after various CM treatments for 24 h. *p < 0.05; **p < 0.01; ***p < 0.001; ****p < 0.0001; ns: no significant difference

The cell-migrating capability of SDF-1α released from PMEZE/mEV scaffolds was assessed using a Transwell migration assay with ADSCs. In the schematic illustration for the experimental design, ADSCs were incubated with medium in the top chamber, and the medium in the bottom chamber was supplemented with PMEZ, PMEZE, and PMEZE/mEV scaffolds, respectively (Fig. 4A). After 24 h incubation, migratory responses of ADSCs were significantly enhanced in the PMEZE/mEV group compared to the other two scaffolds (Fig. 4B and C). Other bioactive materials on the scaffold did not promote ADSC migration, except for mEVs. Moreover, no critical difference was detected between PMEZE/sEV and PMEZE/mEV, indicating that the controlled release of SDF-1α from sEVs and mEVs is effective in the stem cell recruit (Fig. S3). HK2 cells are an immortalized proximal tubule epithelial cell line from normal adult human kidneys and are widely used to prove kidney regenerative activities in vitro [39, 40, 56]. The regeneration-related bioactivities of EDV and mEVs released from the scaffolds were evaluated by comparing the degree of wound healing and cellular proliferation in HK2. EDV is an oxygen radical scavenger that can increase neural stem cell growth in the dentate gyrus after neuronal injury [57]. Moreover, EDV is crucial to wound healing in vivo [58, 59]. Similarly, wound closure rates were significantly accelerated with EDV and mEV addition compared to PMEZ scaffolds (Fig. 4D and E). Besides, HK2 cell viability was more upregulated with the introduction of additional EDV and mEV, although PMEZ increased cell viability by bioactive substances, including ZnO-ALA (Fig. 4F). To demonstrate the strong angiogenic properties of EDV and mEVs, the angiogenesis-related tube formation assay was conducted with HUVECs. Results showed that tube formation was facilitated in PMEZE and upregulated in PMEZE/mEVs. With the EDV-based angiogenic property of PMEZE [60], mEVs also indicated a synergistic angiogenic activity on scaffolds (Fig. 4G). All parameters related to tube formation, such as number of branch points, total tube length, nb junction, and total branch length, gradually increased with EDV addition and mEVs based on PMEZ scaffolds (Fig. 4H; Fig. S4). A similar trend was shown in RT-PCR compared to the expression of angiogenic factors. Vascular endothelial growth factor *(*VEGF) and angiopoietin-1 (ANG-1) are major angiogenic factors in vascular development [61]. Results showed significant upregulation of mRNA expression levels of representative proangiogenic genes, VEGF and ANG1, in HUVECs incubated with PMEZE/mEV scaffolds (Fig. 4I). Collectively, these results suggested the synergistic effects of EDV and mEVs in promoting tissue regeneration. EDV promotes the GDNF/RET neurotrophic signaling pathway in functional mRNA-induced motor neurons and the mouse spinal cord [17]. In particular, GDNF/RET signaling regulates UB formation and branching morphogenesis [18]. To investigate the activities of EDV via the GDNF/RET pathway, various CM were collected from HK2 cells and added to the culture medium of ADSCs at 50% concentration. Four CM were used as follows: CM from HK2 without EDV and mEVs (CM), HK2 treated with mEV for 24 h (mEV-CM; mEV), HK2 treated with EDV for 24 h (EDV-CM; EDV), and HK2 treated with mEV and EDV for 24 h (mEV/EDV-CM; mEV/EDV; Fig. 4J). This study examined whether scaffold incorporated EDV can regulate the GDNF/RET pathway of ADSCs by examining the gene expression of related markers. GDNF and RET gene expression was upregulated in EDV and mEV/EDV groups compared to CM (Fig. 4K). Target genes for downstream GDNF and RET, ETV4 and ETV5, which are required for kidney branching morphogenesis [62], were also promoted in EDV and mEV/EDV groups compared to when they were cultured in mEV-CM (Fig. 4L). These data suggested that EDV could improve the regenerative capacity by activating the GDNF/RET pathway and demonstrated that this efficacy is maximized by the synergistic effect of EDV and mEV. However, this needs to elucidate the precise mechanism of how EDV regulates the GDNF/RET signaling for kidney tissue regeneration in further studies.

### In vivo structural and functional recovery of kidney tissues

**Fig. 5:**
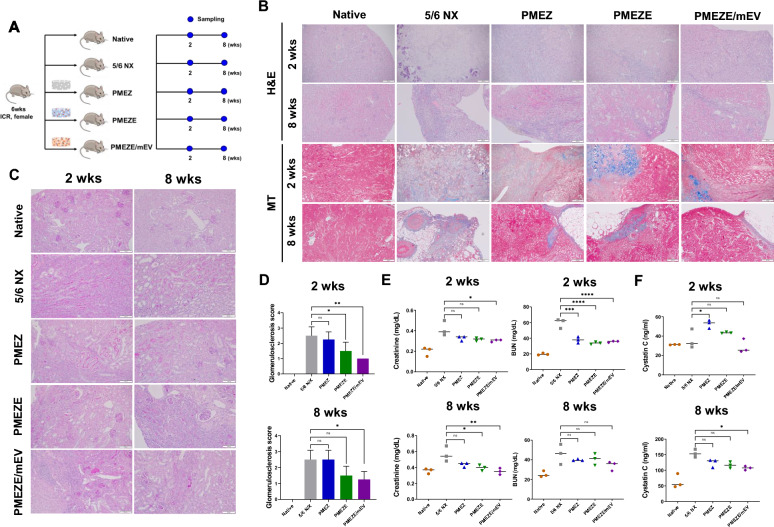
In vivo evaluation for kidney functional restoration capabilities of scaffolds. **A** Schematic diagram of the experimental schedule utilizing the 5/6 nephrectomy mice model. **B** Representative images of H&E and MT staining for evaluation of inflammation and fibrosis in the scaffold region 2 and 8 weeks after implantation, respectively. **C** Representative PAS staining of scaffold region and **D** quantitative analysis to assess glomerulosclerosis 2 and 8 weeks after implantation. Scale bars = 100 μm. **E** Creatinine and BUN levels in the serum 2 and 8 weeks after implantation. **F** Cystatin C level 2 and 8 weeks after implantation. *p < 0.05; **p < 0.01; ***p < 0.001; ****p < 0.0001; ns: no significant difference

With promising results from the biological activities of PMEZE/mEV scaffolds in vitro, four scaffolds were implanted into nephrectomy models to determine the multifunctionalities of novel scaffolds on tissue regeneration and functional kidney restoration. Based on our previous results for kidney regenerative activities of scaffolds containing various engineered EVs, such as tumor necrosis factor-α (TNF-α)/interferon-γ (IFN-γ)-primed MSC-derived extracellular vesicles (TI-EVs), differentiating extracellular vesicles (dEV), and melatonin-modulated extracellular vesicles (mEVs), we aimed to demonstrate the kidney regenerative effects of scaffolds supported with EDV and mEVs in the 5/6 nephrectomy model [39, 40, 50]. The 5/6 nephrectomy mouse model mimics severe renal failure with loss of kidney function in humans and has been commonly used to study CKD [63]. All animal experiments were conducted with five groups, and parameters related to regenerative bioactivities were evaluated at 2 and 8 weeks after scaffold implantation in injured kidney tissues (Fig. 5A). H&E staining showed that the 5/6 NX group led to severe morphologic lesions characterized by severe tubular injury and necrosis (Fig. 5B). In contrast, injured kidneys implanted with PMEZE/mEV scaffolds exhibited notable attenuation of these pathological lesions. Stokman et al. suggested that SDF-1 is not only tissue repair by mediating the trafficking of circulating stem cells to the site of peripheral injury in tissues but also mediates renal repair after ischemia/reperfusion (I/R) injury, which is independent of hematopoietic stem cells (HSC) cell migration [64]. It is possible that SDF-1α derived from sEVs directly and/or indirectly facilitated kidney tissue recovery. However, further investigation is needed to prove that stem cells around the damaged site were recruited by sEVs in the in vivo system. From the MT analysis, the extensive collagen formation of the 5/6 NX group was significantly downregulated after the implantation of PMEZE/mEV scaffolds with regeneration kidney tissues. In detail, reduced collagen deposition was detected in the group of scaffolds with EDV and mEV, indicating synergistic bioactivities of both emerging materials. The morphology of glomeruli in the five groups was examined by PAS staining to analyze functional glomerular tissue regeneration (Fig. 5C). The glomerulosclerosis score was used to evaluate the scarring of the filter system that causes tissue injury and decreased with the implantation of PMEZE/mEV scaffolds (Fig. 5D). These findings indicated that the scaffolds combining EDV and mEVs based on PMEZ scaffolds ameliorated histopathological impairments compared to PMEZ scaffolds. Clinical changes in kidney function were determined by measuring serum creatinine and blood urea nitrogen (BUN) levels, where high levels indicate clinical signs of accumulation of nitrogenous wastes [65, 66]. Biochemical evaluations proved that both factors showed significantly lowered levels with EDV and mEVs incorporation into PMEZ scaffolds; in particular, the creatinine level was similar to that of the native group at 8 weeks after scaffold implantation (Fig. 5E). The C-reactive protein (CRP) level, used as an indicator of acute inflammation via severe infection, injury, and/or chronic disease, was not completely inhibited in all groups but significantly recovered in the PMEZE/mEV group at 8 weeks (Fig. 5F). Taken together, these data indicated that the synergistic effect of EDV and mEVs mitigates kidney damage.

### In vivo evaluation of PMEZE/mEV scaffolds for kidney regeneration-related bioactivities

**Fig. 6:**
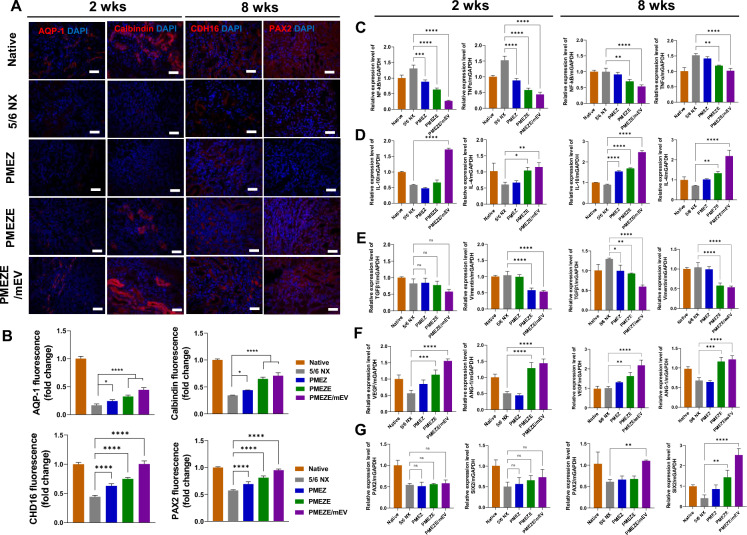
In vivo assessment of kidney regeneration-related bioactivities of the scaffolds. Immunofluorescence images of **A** AQP-1, calbindin, CDH16, and PAX2 2 and 8 weeks after implantation. Blue and red signals indicate nuclei and tubule-related proteins, respectively. Scale bars = 100 μm. **B** Quantitative analysis of relative fluorescence intensity for AQP-1, calbindin, CDH16 and PAX2. Gene expression analysis of the scaffold implanted region by RT-qPCR of **C** proinflammatory, **D** anti-inflammatory, **E** fibrosis, **F** angiogenesis, and **G** kidney regeneration-related markers. **p < 0.01; ***p < 0.001; ****p < 0.0001; ns: no significant difference

To unravel the functionalities of bioactive materials in scaffolds for the 5/6 nephrectomy model, kidney tissues were immunocytochemically stained with various kidney regeneration-related genes. Especially, EDV was expected to facilitate kidney regeneration by upregulating tubule regenerative activities. To identify tubule-related regeneration of EDV-containing scaffolds, representative tubule markers, aquaporin-1 (AQP-1) and cadherin-16 (CDH16) for proximal tubules [67] and calbindin [68] and PAX2 [69] for distal tubules, were selected and observed in injured sites with scaffold implantation. The fluorescent signal of AQP-1 and CDH16, showing negligible signals with nephrectomy, started to be exposed in the PMEZ group, which was maximized by introducing mEVs (Fig. 6A; Fig. S5). Same trends were shown in quantitative data from immunofluorescent images (Fig. 6B). These supported the role of EDV for kidney tissue regeneration by activating tubule formation and the synergistic effects of EDV and mEVs for promoting kidney tissue regeneration. The effects of EDV and mEVs were investigated by quantitative evaluation of the gene expression level related to kidney tissue regeneration at 2 and 8 weeks. In PMEZE/mEV scaffolds, the expression of inflammation-related genes, such as nuclear factor kappa B (NF-κB) and tumor necrosis factor alpha (TNF-α), decreased, whereas the expression of anti-inflammatory genes, such as interleukin (IL)-10 and -4, increased (Fig. 6C and D). Specifically, mEVs play a critical role in regulating inflammation-related factors. Consistent with previous MT staining results, fibrosis-related factors, TGF-β and vimentin, were dramatically reduced in EDV and mEV addition compared to the injured group (Fig. 6E). The expression levels of angiogenesis-related genes, such as VEGF and ANG-1, started to be upregulated in PMEZ groups during the initial time, with ZnO-ALA release, and were maximized with EDV and mEV incorporation for a long period (Fig. 6F). The expression of paired box gene 2 (PAX2) and six homeobox 2 (SIX2) was significantly upregulated in PMEZE/mEV scaffolds at 8 weeks, although there were no significant group-specific differences at 2 weeks (Fig. 6G). The increased expression levels of kidney development-related factors, PAX2 and SIX2, especially at 8 weeks after scaffold implantation, supported that the combination of sEVs and dEVs in PMEZE scaffolds may recruit surrounding MSCs and induce differentiation into kidney progenitor cells, resulting glomeruli regeneration and functional restoration. The property for MSC recruitment of sEV is based on the SDF-1/CXCL4 axis-mediated MSC recruitment activity [70, 71]. These results demonstrated that PMEZE/mEV scaffolds have beneficial effects on kidney repair through inhibition of fibrosis and inflammation and promotion of angiogenesis. However, further studies are needed to unravel the precise mechanism for functional recovery through tissue regeneration.

## Conclusion

Biocompatible PMEZE/mEV scaffolds with EDV and mEV incorporation based on PMEZ scaffolds containing MH-RA, ECM, and ZnO-ALA were developed with PLGA for structural reconstruction and functional recovery of the kidney in the 5/6 nephrectomy model. The overall results of comprehensive experiments demonstrated that PMEZE/mEV scaffolds could enhance angiogenesis and anti-inflammatory abilities, leading to accelerated kidney repair. These scaffolds may promote the regenerative capacity of kidneys by activating the GDNF/RET signaling pathway using EDV. Furthermore, released mEVs, the combination of sEVs and dEVs, could recruit surrounding stem cells to the injured area and accelerate kidney reconstruction via differentiation into kidney progenitor cells. Consequently, these features make PMEZE/mEV scaffolds behave much better in repairing kidney damage than PMEZ scaffolds (i.e., without EDV and mEV) and PMEZE scaffolds (i.e., without mEV). Based on these results, this bioactive scaffold system is a highly motivated strategy for kidney regeneration, offering potential emerging approaches for improving the repair of kidney dysfunction.

## Supplementary Information

Additional file 1.

## Data Availability

The datasets used and/or analysed during the current study are available from the corresponding author on reasonable request.
