# Supplementary material for: Multifunctional extracellular vesicles and edaravone-loaded scaffolds for kidney tissue regeneration by activating GDNF/RET pathway
Source: Nano Converg. 2024 Oct 26;11:43. doi: 10.1186/s40580-024-00450-5 (PMC11512987; doi:10.1186/s40580-024-00450-5)
Supplement: Supplementary file 1 — Additional file 1. [file 40580_2024_450_MOESM1_ESM.docx]

**Figure S1.** Cell viability determined by CCK-8 assay. The cell viability of ADSCs treated with different EDV concentrations (0, 100, 200, 300, and 400 μM) for 24 h. **p* < 0.05, ***p* < 0.01, ****p*<0.001.

**Figure S2.** Degradation rate of the PMEZE scaffolds in a physiological condition (pH = 7.4).


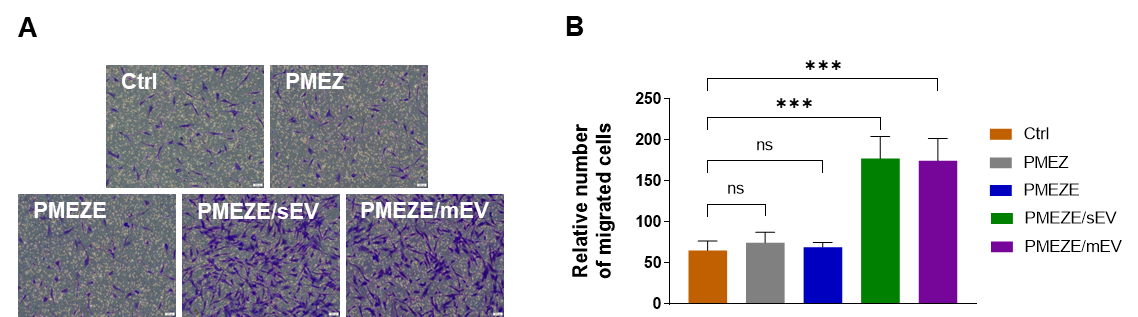


**Figure S3.** *In vitro* analysis for ADSCs migration via SDF1α-overexpressed EV. (A) Microscopy images and (B) quantitative analysis of crystal violet stained ADSCs that migrated from the top chamber to the bottom chamber in response to sEV and mEV. Scale bars = 200 μm.


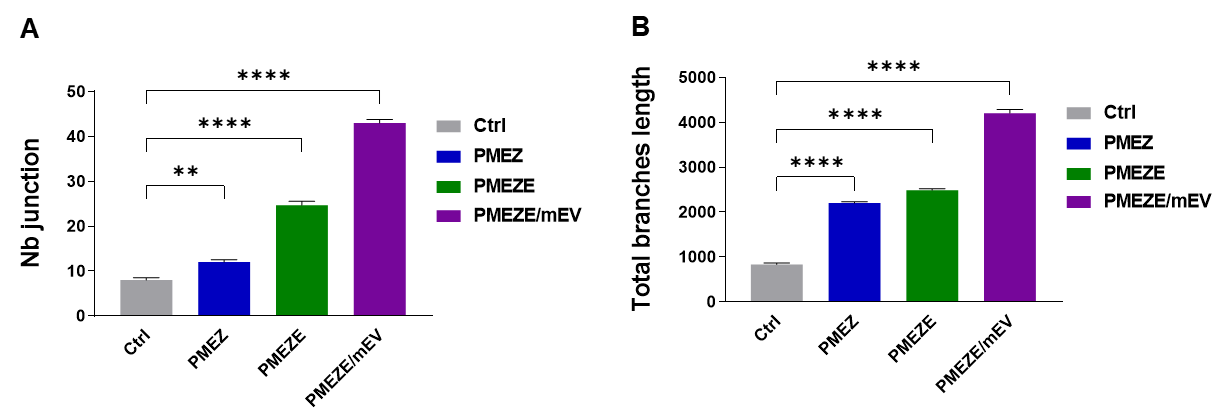


**Figure S4.** Quantitative analysis of tube formation assay characterizing (A) nb junction and (B) total branches length.


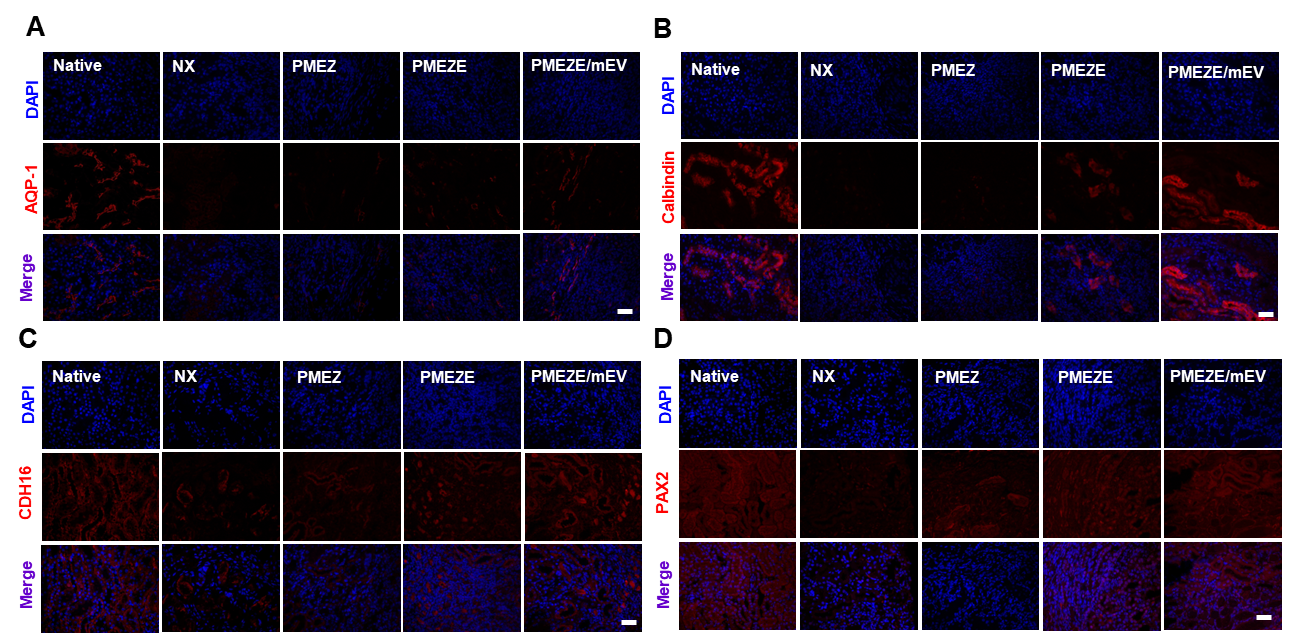


**Figure S5.** Immunofluorescence images of the (A) AQP-1, (B) Calbindin, (C) CDH16, and (D) PAX2 in injured areas. Red; Target proteins, Blue; nuclei . Scale bars = 100 μm.
